# Low-Dose High-Pitch CT Angiography of the Supraaortic Arteries Using Sinogram-Affirmed Iterative Reconstruction

**DOI:** 10.1371/journal.pone.0099832

**Published:** 2014-06-11

**Authors:** Dietrich Beitzke, Richard Nolz, Sylvia Unterhumer, Christina Plank, Michael Weber, Rüdiger Schernthaner, Veronika Schöpf, Florian Wolf, Christian Loewe

**Affiliations:** 1 Department of Biomedical Imaging and Image-guided Therapy, Division of Cardiovascular and Interventional Radiology, Medical University Vienna, Vienna, Austria; 2 Department of Biomedical Imaging and Image-guided Therapy, Division of Neuroradiology and Musculoskeletal Radiology, Medical University Vienna, Vienna, Austria; University of Groningen, Netherlands

## Abstract

**Objective:**

To prospectively evaluate image quality and radiation dose using a low-dose computed tomography angiography protocol and iterative image reconstruction for high-pitch dual-source CT-angiography (DSCTA) of the supraaortic arteries.

**Material and Methods:**

DSCTA was performed in 42 patients, using either 120 kVp tube voltage, 120 mAS tube current, 2.4 pitch and filtered back projection, or 100 kVp tube voltage, 100 mAs tube current, 3.2 pitch, and sinogram affirmed iterative reconstruction. Measurements of vessel attenuation, of the contrast-to-noise ratio (CNR) and the signal-to-noise ratio (SNR) were performed to objectively evaluate image quality. Two readers evaluated subjective image quality and image noise, using a four-point scale. Effective dose was used to compare the differences in radiation dose.

**Results:**

Low-dose protocol application showed significantly higher vessel opacification (p = 0.013), and non-significantly higher CNR and SNR values. There was no difference in the subjective image quality and image noise reading between the protocols. Effective dose was significantly lower using the low-dose protocol (1.29±0.21 mSv vs. 2.92±0.72 mSv; p<0.001).

**Conclusion:**

The combined use of reduced tube voltage, reduced tube current, and iterative reconstruction reduces radiation dose by 55.4% in high-pitch DSCTA of the supraaortic arteries without impairment of image quality.

## Introduction

Computed tomography angiography (CTA) of the supraaortic arteries has become a standard imaging technique for arteriosclerotic disease, acute stroke imaging, and for imaging traumatic arterial dissection [1], [2]. Advantages of this technique include the high spatial resolution, the short acquisition times that reduce motion artifacts and venous overlay, and the high accuracy for the detection of stenosis [3]. The ability to image the entire supraaortic vasculature and to combine CTA of the supraaortic arteries with a computed tomography (CT) of the cerebrum and/or a CT perfusion protocol leads to broad clinical use of this method during the last several years [4]. In 2006 computed tomography was accountable for about 25% of the annual per-capita effective radiation dose in the US [5]. Although until now there is no clear evidence about cancer induction through cumulative radiation doses of below 100 mSv, radiation exposure from medical imaging has drawn widespread attention within the last years [6]. Recent advances in computed tomography have introduced new possibilities for significant radiation dose reduction, including iterative reconstruction algorithms and the introduction of high-pitch scanning. The efficacy and usefulness of radiation dose reduction by using reduced tube voltage and reduced tube current settings have already been reported, but this technique leads to increased image noise, which may alter diagnostic image quality if using filtered back projection (FBP) for image reconstruction [7]. Therefore, the use of FBP in low dose CT scanning is limited, as increased image noise is associated with and reduced image quality [8]. To overcome this limitation and to provide diagnostic image quality even at low dose, iterative reconstruction algorithms for image noise correction may be used [9], [10]. These reconstruction algorithms allow for image noise reduction during postprocessing, as loop-wise comparison and noise correction to the raw data images are performed [11].

Sinogram-affirmed iterative reconstruction (SAFIRE) is a raw data-based iterative reconstruction algorithm that is based on a noise modelling technique. SAFIRE tries to discover picture building and non-picture building pixels during image postprocessing. Therefore SAFIRE utilizes the known propagation of signal and noise in projection data to separate information from noise. With each iterative step, the noise content is determined and subtracted from the image data-set. After each reconstruction loop (also called iteration) the resulting image is then compared to the initial raw data for image validation. The resulting CT data may be considered as a “noise free” base and additive statistical noise. The number of iterations determines the level of noise reduction and image smoothing [9], [11], [12].

In addition, the latest generation of dual-source scanners, can be used high-pitch scanning with a maximum pitch of 3.2. In single-source CT the maximum pitch is usually limited to <1.5. Single source scanning with a pitch above 1.5 will result in sampling gaps. In high-pitch scanning two separated 128-slice detectors are operated with similar energy at a rotation time of 0.28 sec and a table feed up to 46 cm/sec. This allows for gapless scanning at higher pitch values as the second detector is used to fill up the gaps [13]. In various studies that focused on the coronary arteries and other scan regions, this scanning technique confirmed the possibility of significant dose reduction without the loss of diagnostic imaging quality [14], [15]. Recent literature also showed lowered radiation dose values by using the high-pitch mode at CTA of the supraaortic arteries [16].

The purpose of this study was to evaluate image quality and radiation dose of a low-dose, dual source, single energy CTA protocol of the supraaortic arteries using a reduced tube voltage, a reduced tube current, and iterative reconstruction algorithms in high pitch scanning, and to compare this protocol to the standard institutional DSCTA protocol using FBP in order to prove the possibility of significant dose reduction while maintaining image quality [10].

## Materials and Methods

### Patient Population

The study has been approved by the local ethics committee at the Medical University of Vienna, Austria (Local IRB NR: 127/2011). All subjects gave written and informed consent prior to study inclusion. Forty-two consecutive patients with suspected or previously diagnosed vascular disease of the supraaortic arteries and referred for CTA of the supraaortic arteries were enrolled in this prospective, randomized study and assigned to one of the studies groups using a computer-generated randomization list (Table 1). Indications for CTA included stenosis of the subclavian artery (n = 5), stenosis of the brachiocephalic trunk (n = 2), stenosis of the common carotid artery (n = 1), suspected stenosis of the internal carotid artery (n = 24), suspected occlusion of the internal carotid artery (n = 2), suspected aneurysm of the common carotid artery (n = 1), suspected aneurysm of the vertebral artery (n = 1), evaluation of the supraaortic vessels prior to axillo-femoral bypass operation (n = 1), assessment of suspected in-stent restenosis (n = 4),and suspected arteriovenous fistula (n = 1).

**Table 1:** CT study protocols and patient characteristics. Scan Range/Patients data presented as means±standard deviations (range).

	Standard	Low-dose	p-value
**CT Protocol:**			
CT scanner	Somatom Definition Flash		
Slice collimation	2×64×0.625 mm		
Kilovoltage (kV)	120	100	
Tube current (mAs) ref.	120	100	
Tube current modulation	CareDose4D		
Pitch	2.4	3.2	
Contrast/flow	70 ml Optiray 350 at 5 ml/sec		
Image reconstruction	FBP	SAFIRE (Level 3)	
Scan Range (cm)	42±3.5 (35.8–48.5)	42.1±2.9 (37.4–48.6)	0.83 (n.s.)
**Patients:**			
Sex (female/male)	9/12	7/14	
Age (years)	66.5±12 (43–86)	64.1±11.8 (39–90)	0.69 (n.s.)
Body Mass Index	27.3±4.1 (20,7–36,2)	27.1±2.8(22.1–33.5)	0.49 (n.s.)

Exclusion criteria included renal insufficiency, known allergic reaction to iodinated contrast media, age <18, hyperthyroidism, pregnancy and breast feeding.

### Computed Tomography Angiography Image Acquisition

All examinations were performed using a 128–row, dual-source, multi-slice CT scanner (Siemens Somatom Definition Flash, Siemens Medical Systems, Erlangen, Germany). CTA was performed in a caudocranial direction from the level of the aortic arch to the calvarium. Acquisition parameters were 2×64×0.6 mm collimation and 280 ms gantry rotation time in both groups. Scanner settings for the standard protocol were as follows: 120 kVp tube voltage for both tubes; 120 mAs per rotation tube current time product with tube current modulation turned on (CAREDose 4D); and pitch 2.4. In contrast to the standard protocol, the scanner settings for the low-dose protocol were as follows: 100 kVp tube voltage for both tubes; 100 mAs per rotation tube current time product with tube current modulation turned on; and pitch 3.2 (Table 1). Scanner settings for the low dose protocol were based on previous reports in the literature and adopted to the second generation dual source scanner used in our study [7], [16], [17]. For the low dose groups 100 kVp tube voltage was chosen as previous reports using 80 kVp without SAFIRE were associated with increased image noise at the level of the shoulder belt [7]. To achieve further radiation dose reduction in the low dose group, reference tube current settings were lowered to 100 mAs per rotation.

Contrast agent injections were performed using a programmable power injector (OptiVantage, Digital Injection System, Liebel-Flarsheim Company, Cincinnati OH, USA). Optiray, a non-ionic iodinated contrast medium, was used at a concentration of 350 mg iodine/mL for all CTA studies in both groups (Optriray 350, Covidien, Dublin, Ireland).

An eighteen- (40 Patients) or 20-gauge (2 Patients) intravenous catheter was placed, wherever applicable, into the right antecubital vein for the injection of contrast agent. In three patients, the catheter had to be placed into the left antecubital vein because adequate venous access could not be provided on the right side. Bolus tracking technique was used for scan timing, with the reference image at the level of the descending thoracic aortic artery and a threshold for aortic enhancement of 150 HU. Despite using the high pitch mode, the post-threshold delay was set to 5 s in all examinations in order to avoid venous contrast agent remnants and venous contrast agent reflux at the upper thoracic outlet. Seventy mL of contrast material was injected at a constant injection rate of 5 mL/s in patients with an 18 gauge catheter and 4 ml/sec in the two patients with a 20 gauge catheter. All injections were followed by a saline flush of 40 ml.

### Image Reconstruction

All images were reconstructed at 1 mm and 3 mm slice thicknesses. For image reconstruction in the standard group, FBP with a standard medium soft kernel (B31F) was used. SAFIRE with a noise reduction level 3 and the corresponding medium soft kernel for iterative reconstruction I31F was used in the low-dose group.

In addition, standardized curved planar reformats (CPR), as well as maximum intensity projections (MIP), were reconstructed in the coronal and sagittal planes, respectively.

### Objective Image Analysis

A segment based analysis was chosen for the evaluation of objective image quality. Intravascular attenuation values (Hounsfield Units; HU) for the calculation of the contrast-to-noise ratio (CNR) and signal-to-noise ratio (SNR) were obtained at the level of the aorta, and bilaterally at three different anatomical levels in the course of the carotid arteries, using 3 mm thick axial reconstructed images. Measurements of vessel attenuation were performed within the aortic arch, the common carotid artery 2 cm distal to its origin, at the level of the carotid bifurcation, within the extracranial course of internal carotid artery, in the carotid sinus, and within the M1 segment of the medial cerebral artery. Care was taken to avoid measurements directly next to vessel wall calcifications.

To quantify venous enhancement vessel and reflux of contrast agent into cervical veins, attenuation measurements within the sagittal sinus, the transverse sinus and the jugular veins at the level of the bifurcation were performed.

CT attenuation values for the cervical CNR calculation were obtained from the sternocleidomastoid muscle (at the same level of the vessel measurements), and background noise (BN) was obtained from three areas of the surrounding air (right, left, anterior). As the field of view did not allow BN measurements in all patients at the level of the aorta, the level of the common carotid artery was used for CNR and SNR calculation at the aortic level. The area of the region of interest for HU measurements was kept constant (0.11–0.13 cm^2^) during all measurements. CNR and SNR data were also calculated from the source data of axial 3 mm thick slices and was derived using the following formulas:
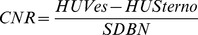


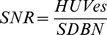
(*HUVes* = attenuation value within the vessel, *HUSterno* = attenuation value within the sternocleidomastoid muscle, *SDBN* = average standard deviation of the surrounding air at right, left, and anterior positions). All data was read in three sessions.

### Subjective Image Quality Analysis

Two readers, not involved in the objective image quality reading and blinded to the protocol, performed the image quality reading independently, using a four-point scale:

Grade 0 (non-diagnostic): vascular segments cannot be assessed because of streak artifacts, vascular contrast, or graininess of the images.

Grade 1 (poor-diagnostic): patency or occlusion of the vascular segment can be assessed, but image quality is unsatisfactory.

Grade 2 (good quality): good image quality providing all details necessary for an adequate diagnosis.

Grade 3 (excellent): excellent image quality enabling sufficient differentiation of even small structures.

Readers were also asked to rate image noise on a four-point scale (0–3).

Grade 0: no noise.

Grade 1: little noise, natural graininess of the images.

Grade 2: noise above average but no impairment of the diagnostic image quality.

Grade 3: strong image noise with impairment of the diagnostic image quality.

Image quality and image noise scoring was obtained from the following segments: Aortic arch, common carotid artery, carotid bifurcation, internal carotid artery (extracranial course), carotid sinus, medial cerebral artery (M1), origin of the vertebral artery (V1), extracranial course of the vertebral artery (V2–V3) and the intradural course of the vertebral artery (V4). With the exception of the aortic arch, image quality and noise ratings were obtained from the left and right side separately.

### Radiation Dose

For the estimation of radiation dose, the Dose Length Product (DLP) was calculated automatically by the CT scanner system, based on the multiplication of the Computed Tomography Dose Index (CTDI) according to the imaging range. Effective dose (ED) was subsequently calculated by using a standard conversion factor of 0.0051 mSv mGy^−1^ cm^−1^, and as the DLP values provided by the scanner system were based on a 32-cm body phantom, the values were then multiplied by a correction factor of 2.4 to correct for a 16 cm phantom as proposed by Lell et al [18], [19].

### Statistical Analysis

Patient characteristics, imaging range, signal parameters, DLP, and the ED of both groups were analysed using the SPSS statistical package (SPSS Windows, version 20.0; SPSS, Chicago, IL, USA). Metric data, such as age, BMI, or scan length were expressed using means ± standard deviations. Nominal data are presented using percentages. Unpaired t-tests were performed for metric data to assess differences between the study protocols. In order to compare results of 100 and 120 kVp for all arterial and venous segments, two-way mixed model ANOVAs using unstructured covariance matrices were assessed separately, taking multiple measures per patient into account.

Agreement about subjective image quality between the two raters was tested using Cohen's kappa statistics. Image quality and noise ratings between the two study groups were compared using a Mann-Whitney Test. The alpha level for all tests was set at p = 0.05.

## Results

All CT scans were performed without complications according to the study protocol.

### Patient Population

19 female patients and 23 male patients were included in this study. The mean age was 64.3 years (range, 22 to 85 years), and mean body mass index (BMI) was 27.6 (range 19.5 to 36.5). Patient characteristics, including height, weight, and body mass index, were equally distributed within the two groups, with no significant differences between the groups (Table 1).

### Image Quality

#### Objective image quality

**Figure 1:**
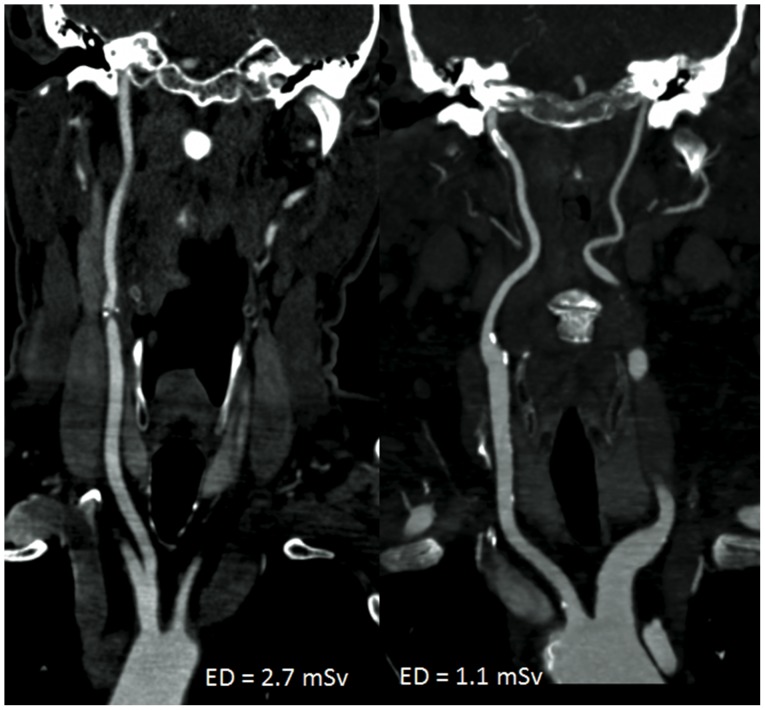
Comparison of image quality in two patients with suspected stenosis of the internal carotid artery. Curved planar reconstructions of (A) the standard protocol and (B) the low-dose protocol demonstrate similar diagnostic image quality in both protocols with a more smoothed appearance and reduced image noise at the level of the common carotid arteries using SAFIRE. Significantly lower radiation dose at B.

**Figure 2:**
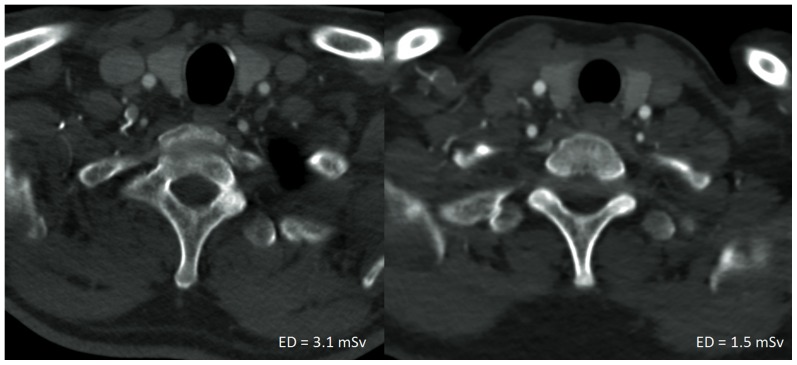
Comparison of image quality at the thoracic outlet. Axial images with similar window setting (W900/L250) using the standard protocol and FBP (A) and at low-dose, using SAFIRE for image reconstruction (B). Both studies were rated having excellent diagnostic image quality. Higher vessel attenuation and reduced image noise is evident at low-dose and SAFIRE (B).

Evaluation of arterial intravascular attenuation values showed significantly higher intravascular HU values at low-dose CTA (p = 0.013) (Fig. 1, 2). Measured HU values within the sternocleidomastoid muscle were significantly higher at low-dose CTA (p = 0.006). BN was non-significantly lower (p = 0.059) at the low dose. Calculated CNR and SNR values showed non-significantly higher values at the low dose (CNR: p = 0.197; SNR: p = 0.152) (Table 2; Fig. 1, 2).

**Table 2:** Group comparison of objective image quality (mean vessel attenuation, muscle attenuation, background noise, CNR, SNR).

	Standard Protocol mean±SD		Low-dose protocol mean±SD		p-value
**Arterial Vesselattenuation (HU)**					
Aorta	362.3±91.48		420.2±75.97		
	Right	Left	Right	Left	
Common carotid artery	358.1±93.3	370.4±115.1	419.1±79.5	423.5±81.1	
Bifurcation	405.3±93.9	417.3±114.2	477.8±91.7	480.7±95.7	
Internal carotid artery	354.2±87.1	370.4±104.9	433.8±78.4	436.3±81.2	
Carotid sinus	362,6±81,5	378,3±103,6	475,4±87,6	462,3±84,8	
Medial cerebral artery	345,5±75,3	349,0±93,8	428,9±81,0	444,9±89,3	
*Overall p*					*0.013* †
**Backgroundnoise** *					
Common carotid artery	6.32±1.9		6.08±1.7		
Bifurcation	5.53±1.5		5.34±0.7		
Internal carotid artery	6.13±1.4		6.12±1.2		
*Overall p*					*0.59*
**Muscle (HU)**					
Aorta	46.7±12.71		50±13.33		
	Right	Left	Right	Left	
Common carotid artery	58.3±14.7	56.3±17.3	70.8±17.8	67.7±21.9	
Bifurcation	58.4±10.1	59.6±11.7	64.8±7.9	61±9.1	
Internal carotid artery	49.5±13.9	50.2±10.7	54.3±16.2	53.3±11.8	
*Overall p*					*0.006* †
**CNR**					
Aorta	55.78±28.40		65.81±23.10		
	Right	Left	Right	Left	
Common carotid artery	53.4±29.6	55.5±36.7	62.2±23.1	63.6±24.3	
Bifurcation	68.1±27.9	70.6±32.7	77.7±19.9	80.5±23.8	
Internal carotid artery	52.6±21.5	58.6±31.9	64.5±19.8	63.6±18.2	
*Overall p*					*0.2*
**SNR**					
Aorta	58.41±27.9		73.11±26.9		
	Right	Left	Right	Left	
Common carotid artery	63.4±32.1	66.1±38.1	74.5±24.9	75.3±26	
Bifurcation	79.3±30.3	82.2±35.2	89.8±20.9	91.9±23.9	
Internal carotid artery	61.1±22.7	67.5±33.6	73.4±20	72.9±20.3	
*Overall p*					*0.15*
**Venous Vesselattenuation (HU)**					
Sagital Sinus	238.5±74.76		256.1±60.2		
Δ to M1 (mean)	108.7		224		
	Right	Left	Right	Left	
Transverse Sinus	194.0±44.15	192.2±47.5	230.8±60.5	221±44.1	
Jugular Vein	126.5±129.7	106.4±34.4	108.2±23.9	102.2±22	
Δto Bifurcation (mean)	278.8	310.9	369.6	378.5	
*Overall p*					*0.121*

Mixed model ANOVA was used for group comparison of objective image quality. Data are means±standard deviations.

HU = Houndsfield units, CNR = contrast-to-noise ratio, SNR = signal-to-noise ratio. BN = Standard deviation HU.

*BN at the level of the aorta = BN ACC.

† = Significance level <0.05.

Mean venous opacification values did not differ between the study groups (p = 0.121). Venous overlay at the level of the sagittal sinus and the transverse sinus was higher at low dose, but at the same time higher attenuation differences between the arterial (M1) and the venous territory (sagittal sinus) were measured at low dose (Table 2). HU values within the jugular veins showed mean attenuation values between 100 and 126.

#### Subjective image quality

Of the 1428 vascular segments in both study groups, 4.1% (n = 58) were occluded, and thus, were excluded from further analysis. Overall image quality ratings and noise ratings did not differ between the protocols (p = 0.4; noise: p = 0.91). The majority of the segments in both protocols were rated to be of excellent image quality (Grade 3), with 86.4% in the standard dose and 86.1% at the low dose group (Fig. 2). There were 10.6% of the segments at the standard protocol and 9.1% of the segments at the low dose that were rated to be of good image quality (Grade 2), and 0.3% (n = 2) of the segments in both protocols were rated to be of poor image quality (Grade 1). Insufficient image quality in these two patients was based on low cardiac output resulting in reduced vessel attenuation at the level of the carotid sinus. The interrater agreement was moderate for image quality ratings (κ = 0.532; CI [0.43; 0.63]) (Table 3).

**Table 3:** Image reading parameters including interrater agreement measures for both protocols.

Image Quality	Standard Protocol		Low-dose Protocol	
0	0.0%	n = 0	0.0%	n = 0
1	0.3%	n = 2	0.3%	n = 2
2	10.6%	n = 76	9.1%	n = 65
3	86.4%	n = 617	86.1%	n = 615
Occluded Segments	2.7%	n = 23	4.5%	n = 36
	*p*		*p = 0.21*	
	*95% CI*		*0.43; 0.63*	
	*Interrater agreement*		*κ = 0.52*	
**Image Noise**	**Standard Protocol**		**Low-dose Protocol**	
0	77.6%	n = 554	75.9%	n = 542
1	19.5%	n = 139	19.3%	n = 138
2	0.3%	n = 2	0.3%	n = 2
3	0.0%	n = 0	0.0%	n = 0
Occluded Segments	2.6%	n = 23	4.5%	n = 36
	*p*		*p = 0.5*	
	*95% CI*		*0.66; 0.79*	
	*Interrater agreement*		*κ = 0.72*	

CI indicates confidence interval.

The evaluation of subjective image noise ratings showed 77.6% rated as having no noise in the standard protocol (Grade 0) and 75.9% of the segments rated as such in the low dose protocol. Moderate noise (Grade 1) was evident in 19.5% of the segments in the standard group and in 19.3% of the segments at low dose. Again, 0.3% of the segments in each group (n = 2) were classified having noise above the average (Grade 2). The two patients with noise ratings above the average presented with BMI values >30 and image noise was located at the level of the thoracic aperture. The interrater agreement was good for the noise ratings (κ = 0.724; 95% CI [0.64; 0.79]) (Table 3).

### Radiation Exposure

**Figure 3:**
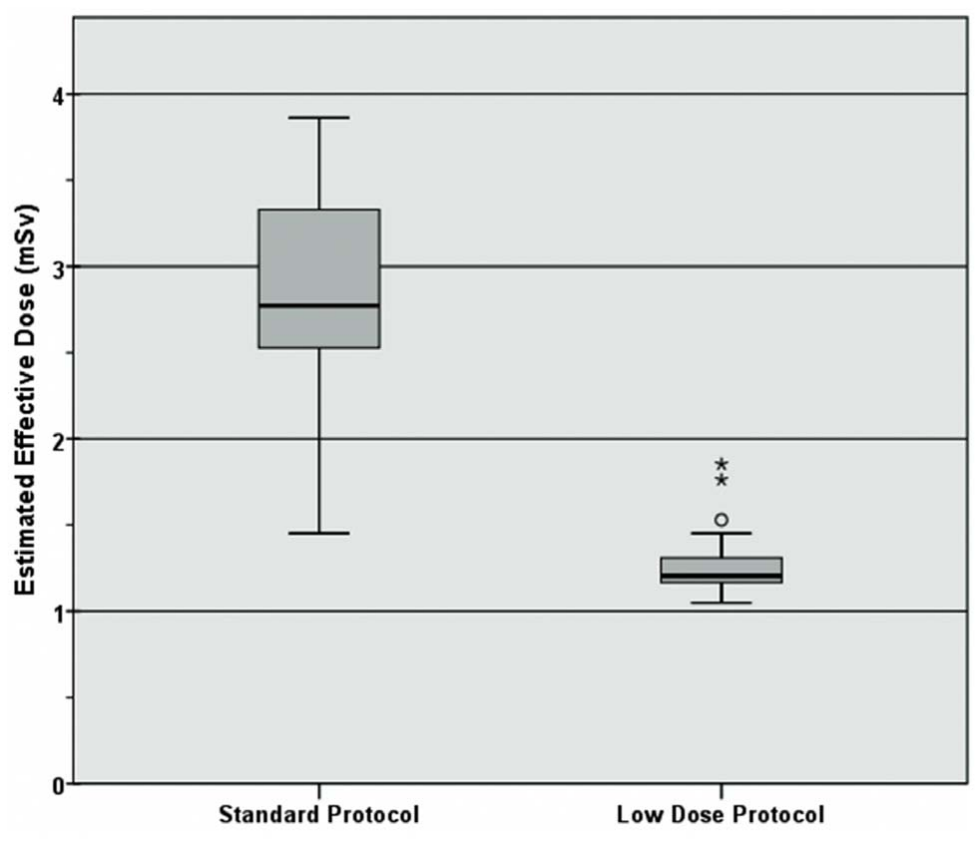
Box-Whisker plots of the estimated mean effective dose (mSv) for the study protocols.

There was no significant difference in the imaging range between the groups (41.8±2.7 cm vs. 42.6±4.7 cm; p = 0.57; Table 1), enabling a comparison of the DLP between the two groups. Mean CTDI was 5.3±1.1 mGy for the standard protocol and 2.4±0.8 mGy at low dose (p<0.001). Mean DLP was 225.4±55.315 mGy*cm in the standard protocol, and 99.2±15.2 mGy*cm at low-dose (p<0.001). Estimated mean ED was 1.21±0.19 mSv for the low dose protocol and 2.76±0.68 mSv for the standard protocol (p<0.001; Table 4; Fig. 3). The latter equals an average reduction in the ED of 55.4%.

**Table 4:** Results from group comparison of radiation dose (unpaired t-test).

	Standard protocol mean±SD	Low-dose protocol mean±SD	p-value
**Radiation Dose**			
CTDI (mGy)	5.3±1.1	2.4±0.8	<0.01
DLP (mGy*cm)	225.4±55.3	99.2±15.9	<0.01
ED (mSv)	2.8±0.7	1.2±0.2	<0.01

Data are means±standard deviations.

CDTI = Computed Tomography Dose Index (mGy); DLP = Dose Length Product (mGy*cm); ED = estimated effective dose (mSv).

## Discussion

In imaging of carotid artery disease invasive angiography as the gold standard has nowadays been replaced by non-invasive imaging modalities like ultrasound (US) magnetic resonance angiography (MRA) and CTA. CTA has a high sensitivity, specificity and accuracy in detection and characterization arteriosclerotic disease of the carotid artery and proofed to be a valuable tool, especially in the acute setting, if there is discordance between US and MRA, or if MRA is contraindicated [3]. With the introduction of new scanner techniques, enormous improvements in CT technique could be observed, including mainly advances in spatial and temporal resolution, making CT applicable for many different indications all through the body. As a consequence, the total number of CT scans performed has increased, and the exposure to radiation doses through CT imaging has become a cause for concern, particularly regarding the potential for cancer induction by CT-associated radiation dose exposure [5], [20].

Different ways to reduce the radiation dose in CT, including scanning at low kV and reduced mAs settings, use of high pitch scanning, and dose modulation techniques, have been evaluated in clinical trials [7], [21], [22]. Using FBP for image reconstruction, most of these techniques are associated with increased image noise, impairing diagnostic image quality at low dose. Recently published studies using iterative reconstruction algorithms on so-called ‘half-dose’ images showed the possibility of an up to 50% dose reduction in CTA, compared to FBP in an intraindividual comparison [23]. Our study evaluated a low-dose protocol for CTA of the supraaortic arteries by combining three different, already established techniques for radiation dose reduction, including low kV settings, reduced mAs settings, and an elevated pitch. To overcome the limitation of increased image noise at low dose, SAFIRE for image reconstruction was applied to the low-dose study group. Our study underlined the possibility of a significant radiation dose reduction of more than 50% at CTA of the supraaortic arteries an unselected patient collective.

Using a lower tube voltage in CTA has two major advantages. First, it is the most effective way to reduce radiation dose. Lowering the tube voltage from 120 to 100 kVp has the potential to reduce the radiation dose applied during scanning by up to 40% [24]. In a recent study 70% dose reduction using 80 kV tube voltage at CTA of the supraaortic arteries using a 64-slice scanner and FBP was reported [7]. However the authors were confronted with streak artefacts and increased image noise at the level of the shoulder belt. Therefore, 100 kVp tube voltage was chosen for our study, as a further dose reduction was achieved by using reduced tube current settings and a high pitch. The second advantage of low kVp settings in CTA is the increase in intravascular contrast due to an increase in intravascular iodine attenuation at low kVp, as the mean photon energy of the x-rays approaches the k-edge of iodine (33.2 keV) [8]. Consequently in our study, significantly higher intravascular attenuation was evident in our low-dose group at 100 kVp (Fig. 1, 2). This effect can positively be used in CTA to either increase the vessel-to-background contrast, and thus, the image quality, or to reduce the total amount of iodinated contrast agents, as shown in previous studies [25], [26]. However, to maintain full comparability of the low-dose protocol to the standard protocol, the total amount of iodinated contrast agent was not changed for the low kVp group in our study.

Since the kVp settings were not lowered to the maximum of 80 kVp to avoid severe artifacts at the level of the thoracic aperture, the tube current time product was lowered by 20% as an additional radiation dose reduction strategy in our study. SAFIRE was successfully used in the low-dose group to eliminate potentially upcoming image noise at low dose. Several published studies using lowered kVp settings in abdominal, pulmonal and supraaortic CTA showed significant higher BN or image noise in their results [7], [27], [28]. Contrary to these reports, the application of SAFIRE in the low-dose group resulted in a tendency toward lower background noise and non-significantly higher CNR und SNR values in the study group. This shows that the expected increase in image noise at low dose was completely compensated by the application of iterative reconstructions providing similar image quality while saving half of the radiation dose. Especially at the origin of the vertebral artery and within the carotid sinus, representing difficult to image areas in CTA of the supraaortic arteries, no differences were observed between the study groups. The results of our prospective study go along with reports in the literature describing the effectiveness of iterative reconstruction on so called “half dose images” in abdominal CT or in CTA of the thoracic aorta [9], [23].

Results from the subjective image quality reading are probably even more important for the evaluation of low-dose protocols and to test their practicality in the clinical practice, as vessel attenuation is not the only important factor in preserving diagnostic image quality at low-dose. The scan range for CTA of the supraaortic arteries includes the bony structures of the upper thoracic aperture and the skull base, two anatomic areas that can potentially interfere with low-dose scanning [29]. Despite this, results from the subjective image quality rating in our study completely concur with results from objective image quality calculations. Both readers rated 86% of all vascular segments in both study groups as having excellent image quality. The interrater agreement concerning image quality was moderate, which may be explained by the more smooth appearance of the CT images reconstructed with SAFIRE. These images may appear “unnatural” to radiologists trained in FBP. There was also no difference in the rating of noise between the readers, and interrater agreement in noise ratings was good. These findings are different to previous work, reporting on increased image noise at low kVp without the use of SAFIRE [7]. Our results could be achieved in a clinical representative patient collective including obese patients and/or patients presenting with vascular pathologies, mainly stenosis. In our study Level 3 of SAFIRE was used in order to avoid an exaggeratedly smooth appearance of the low-dose CT images. If the noise reduction level is set too high or images at standard dose are postprocessed with iterative reconstruction algorithms the images will become blurred. If the noise reduction is set too low on the other hand, image noise remains and may result in impaired diagnostic image quality at low-dose.

Streak artifacts due to dense contrast agent remnants within the veins at the upper thoracic outlet may also alter diagnostic image quality in CTA of the supraaortic arteries, as neighbouring arteries become obscured [30]. These artifacts may be prevented by the application of the contrast agent into a right-sided cubital vein. However, if this is not possible, or in case of insufficient contrast agent mobilisation (e.g. heart failure), iterative reconstruction may also be useful in the reduction of these artifacts.

Beside the above described advantages of iterative reconstruction in CTA there may also be some pitfalls. Based on the functionality of SAFIRE by small hypodens structures adjacent to very hyperdens structures may be interpreted as non-picture building pixels during image reconstruction and may be filtered during iterations. In question of in-stent restenosis or intimal hyperplasia this may theoretically false negative results if dealing with small vessel and stent diameters or in case of severe beam hardening artifacts from the stent material [11]. On the other hand there are first reports about an improved instent visibility in cardiac CTA by using raw data based iterative reconstruction in cardiac CTA [31], [32].

The last adaption of the image acquisition protocol for dose-saving attempts was the use of a pitch factor of 3.2 in the study group, compared to 2.4 in the reference group examined with the standard protocol. Application of the high-pitch mode reduces acquisition of overlapping slices, as two radiation sources allow for gap free scanning at a high table feed. Additionally short scanning times also reduce radiation exposure to the patient [33]. In an evaluation of various pulmonary CTA protocols including 100 kVp settings at high pitch De Zordo et al. showed significant lower DLP values using the high pitch mode in DSCTA in comparison to single source CTA at 100 kVp at comparable tube current settings [34]. Lowest dose was found at 100 kVp and high pitch in this study. In a recent published study by Korn et al. high-pitch DSCTA showed 35% lower radiation dose values compared to single source low-pitch CTA of the supraaortic arteries [35]. In both study groups 120 kV tube voltage was used. In our study, a high pitch was used with both groups (2.4 vs. 3.2), and, from our data, we are not able to clearly estimate the contribution of the difference in pitch to the overall scan dose-saving. But, as a 2.4 pitch represents our approved standard institutional CTA protocol, it was not changed for study purposes in order to avoid higher radiation doses for the reference group by reducing the pitch factor in the study. An additional benefit of using the high pitch mode is the fast scanning time reducing the likelihood of motion artifacts during the scanning in restless and pediatric patients [36].

Venous overlay is another important aspect in CTA of the supraaortic arteries [30]. While using the high pitch moderate venous enhancement of 200 to 250 HU was evident at the intracranial sinuses in both groups but no essential enhancement was measured within the internal jugular veins in both groups. Therefore high pitch scanning with bolus tracking enables to capture the bolus mainly within the arterial system and the intracranial sinuses and disturbing venous overlay within the cervical veins can be avoided.

Some limitations of our study have to be addressed. An intra-individual comparison to exclude hemodynamic factors of vessel attenuation was not performed due to ethical considerations. No direct comparison to a single source CT scanner was performed, as all scans in our institution are performed at dual source. However, from the literature ED values ranging from approximately 4 to 6 mSv are described for standard single source CT, showing even much lower dose in our standard group compared to single source low pitch CT [4], [7]. No comparison to the gold standard invasive angiography was performed. Finally, although more than 1400 vascular segments were analysed in this study, a larger sample size may have detected differences in subjective and objective image reading between the study groups.

In conclusion, iterative reconstruction algorithms enable a significant radiation dose reduction by deleting the increased image background noise at lowered tube voltage and tube current settings during image postprocessing. Image quality did not differ between our study protocols, with an even greater intravascular attenuation at low dose. Therefore, the use of 100 kVp tube voltage, reduced tube current, a high pitch, and the use of sinogram-affirmed iterative reconstruction algorithms in CTA of the surpaaortic arteries enables a radiation dose reduction of more than 50% in an unselected patient collective without the loss of diagnostic image quality.
